# Empathy and bystander helping behavior in cyberbullying among adolescents: the mediating role of internet moral judgment and the moderating role of internet self-efficacy

**DOI:** 10.3389/fpsyg.2023.1196571

**Published:** 2023-09-05

**Authors:** Yang Hu, Tian Zhang, Hui-fen Shi, Cui-ying Fan

**Affiliations:** ^1^Key Laboratory of Adolescent Cyberpsychology and Behavior, Ministry of Education, School of Psychology, Central China Normal University, Wuhan, China; ^2^School of Preschool Education, Hubei Preschool Teachers College, Wuhan, China

**Keywords:** empathy, cyberbullying, bystander helping behavior, internet moral judgment, internet self-efficacy

## Abstract

**Introduction:**

Cyberbullying poses a significant challenge among adolescents. If bystanders stand up and help victims, their helping behavior may be able to reduce the frequency and negative impact of cyberbullying. This study investigates the association of empathy, internet moral judgment, and internet self-efficacy with bystander helping behavior among adolescents, building upon the empathy-altruism hypothesis, bystander intervention model, and dual-process model of morality.

**Methods:**

A sample of 919 Chinese adolescents from 3 schools in Hunan, Jiangxi and Guangdong provinces completed the Basic Empathy Scale, Internet Moral Judgment Questionnaire, Internet Self-Efficacy Questionnaire and Styles of Bystander Intervention Scale. And we constructed a moderated mediation model to examine the relationship between empathy and bystander helping behavior in cyberbullying and assessed the mediating role of internet moral judgment and the moderating role of internet self-efficacy.

**Results:**

Our findings revealed a significant positive correlation between empathy and bystander helping behavior in cyberbullying. Internet moral judgment mediated the relationship between empathy and helping behavior, whereas internet self-efficacy moderated the latter half of the mediation pathway. Specifically, the association between internet moral judgment and helping behavior was stronger for bystanders with higher levels of internet self-efficacy compared with those that have lower levels.

**Discussion:**

These results further deepen our understanding of the mechanisms involved in bystander helping behavior in cyberbullying, thus providing a basis for future interventions to encourage more helping actions from bystanders during cyberbullying incidents.

## 1. Introduction

Cyberbullying refers to the act of an individual or group using electronic means of communication to harm repeatedly individuals who are less capable of defending themselves (Smith et al., 2008). Studies have shown that approximately 17–38% of adolescents have engaged in some form of cyberbullying (Yang et al., 2018; Chu et al., 2018b), especially among eighth-grade students, where the incidence of cyberbullying is higher (Williams and Guerra, 2007).

Adolescents who experience cyberbullying victimization may face a range of negative outcomes, including emotional distress, anger, sadness (Ybarra, 2004; Topcu et al., 2008; Patchin and Hinduja, 2016), social anxiety (Juvonen and Gross, 2008), depression (Schneider et al., 2012; Chu et al., 2018a), and diminished self-esteem (Katzer et al., 2009). Given the high prevalence and negative consequences of cyberbullying among adolescents, it is crucial for schools to implement appropriate intervention strategies.

As with traditional bullying, there are three primary roles in cyberbullying scenarios: the bully, the victim, and the bystander. However, the existing literature primarily addresses the roles of bullies and victims, largely neglecting the bystander’s role in cyberbullying (Huang et al., 2019). It is important to recognize that a substantial number of adolescents have witnessed cyberbullying, making the role of the bystander the most common among the three. For instance, 88% of American adolescents have reported witnessing cyberbullying on social media (Lenhart et al., 2011), and the proportion of bystanders among Malaysian youths aged 17 to 35 years increased from 61 to 70% within 2 years (Balakrishnan, 2015, 2017). Bystanders are the largest group involved in cyberbullying, mainly owing to the open and anonymous nature of the online environment, which results in a considerable number of potential audience members in cyberbullying situations (Kowalski et al., 2014). Studies have shown that bystanders who stand up to help a victim in an online setting can effectively suppress bullying behavior and reduce or alleviate the harm of bullying to the victim (Jones et al., 2015). Crucially, it can also prompt other bystanders to adopt stronger control and normative beliefs in helping victims (Salmivalli, 2010).

Nonetheless, the research by Song and Oh (2018) reveals that 69.4% of bystanders fail to take any intervention measures when confronted with cyberbullying incidents. Consequently, this study focuses on the helping behaviors of bystanders in cyberbullying events and further explores the factors associated with such behaviors. The aim is to recommend constructive intervention approaches that encourage more students to become defenders among cyberbullying bystanders, ultimately decreasing the prevalence of cyberbullying incidents.

The empathy–altruism hypothesis (Batson et al., 1991) asserts that empathy is the basis for altruistic actions. When individuals with high empathy witness others in distress, they are more likely to experience the suffering of the person in distress and feel sympathy and compassion for such individuals, which, in turn, makes them engage in helping behaviors (Ji, 2021). Previous studies have shown that empathy was associated with helping behavior (Freis and Gurung, 2013; Van Cleemput et al., 2014; Erreygers et al., 2016; Zhao et al., 2023). Zheng and Zhao (2015) reveal a significant positive correlation between empathy and online altruistic actions, suggesting that Baston’s empathy–altruism hypothesis is equally relevant in digital environments.

Regarding bystander behavior, Van Cleemput et al. (2014) contend that in cyberbullying situations, bystanders with higher empathy levels are more likely to assist victims. Conversely, those with lower empathy levels may either facilitate bullying or exhibit indifferent, uninvolved behavior. Based on these findings, the first hypothesis of this study is put forward:

*H1*: Empathy is positively associated with helping behaviors among cyberbullying bystanders, and bystanders who are more empathetic demonstrate more helping behaviors toward victims in cyberbullying situations.

Empathy, as a form of moral emotion (Jun and Xiaoxiao, 2011), plays a crucial role in guiding helping and altruistic behaviors, both of which fall under the umbrella of moral behaviors (Wu and Liu, 2014). The dual-process model of morality suggests a direct association between moral emotions and moral behavior and an indirect one through the mediation of moral reasoning (Chaiken and Trope, 1999; Wu et al., 2017). Studies have shown that moral reasoning can partially mediate the impact of moral emotions on online helping behavior (Wu et al., 2017). Moral reasoning involves considering what is wrong about moral behaviors and moral judgment involves evaluating moral behaviors (Tong and Talwar, 2020). According to Haidt (2001), moral reasoning serves to justify an individual’s moral judgments that have already been made. The level of moral reasoning reflects not only the individual’s moral judgment ability but also the consistency between their moral judgments and corresponding moral behaviors (Kohlberg and Candee, 1984). Moral judgment ability refers to an individual’s capacity to make decisions and judgments about morality based on their internal moral principles and to act upon these judgments (Shaogang and Huihong, 2006).

Numerous studies have found that empathy is positively associated with moral judgment (Decety and Cowell, 2014; Patil and Silani, 2014). Empathic responses encourage individuals to feel emotions and pain from the perspective of others, leading them to engage in morally relevant behavior or make more stringent judgments about violations. The deeper an individual’s perception of harm, the higher their moral judgment scores (Wang and Yang, 2021). With the widespread adoption of the Internet and the increase in online activities, Guo-ying (2015) has introduced the concept of internet moral judgment, extending traditional moral judgment into the online context. Internet moral judgment operates similarly to moral judgment in general real-life domains but with slight differences owing to the virtual and anonymous nature of the online environment. Consequently, individuals may demonstrate greater autonomy when making moral judgments in cyberspace and may be less influenced by public opinion and conformity (Fan, 2012; Guo-ying, 2015). This study explores whether empathy is positively associated with an individual’s moral judgment at the cyberspace level in online contexts.

Guo (1999) notes that the higher the development stage of moral judgment, the greater the maturity of moral behavior, indicating that moral judgment is more strongly associated with individual moral actions, with the two exhibiting high consistency. Additional research has shown that moral judgment is positively associated with adolescents’ altruistic prosocial behavior (Eisenberg and Spinrad, 2014; Patrick et al., 2018) and that adolescents with higher moral judgment abilities exhibit more helping behaviors (Li, 2005). Similarly, in online contexts, studies have found a significant positive correlation between college students’ internet moral judgment abilities and online altruistic behavior (Li, 2015). Helping behaviors from cyberbullying bystanders is one example of online altruistic behavior. Thus, it is possible that a positive correlation exists between internet moral judgment and helping behaviors from cyberbullying bystanders. Drawing on the dual-process model of morality and relevant empirical research, the second hypothesis is proposed:

*H2*: Internet moral judgment mediates the relationship between empathy and helping behaviors in cyberbullying bystanders.

When discussing the association of empathy and moral judgment with helping behaviors, it is essential to consider the role of internet self-efficacy. While an individual’s empathy level can impact bystander helping behavior in cyberbullying scenarios through the mediation of internet moral judgment, not all individuals with high internet moral judgment necessarily engage in helping behavior. Latané and Darley (1970) propose the bystander intervention model to elucidate the phased changes in bystander helping behavior. According to this model, when bystanders perceive an event’s urgency, they feel compelled to engage in helping behaviors—a process connected with personal moral factors such as moral judgment. When individuals recognize that an action is immoral, their moral consciousness is activated, increasing the likelihood of them engaging in helping behaviors. However, before doing so, individuals would assess their ability to intervene and determine the appropriate course of action. In this context, self-efficacy plays a significant role (Huang et al., 2019). Research has shown that adolescents with high moral judgment maturity but low self-efficacy might lack the confidence to perform helping actions, indicating that self-efficacy may moderate the association between moral judgment and helping behavior (Comunian and Gielen, 1995). Scholars contend that the bystander intervention model can be similarly applied to explain bystander behavior in cyberbullying incidents (Dillon and Bushman, 2015; Brody and Vangelisti, 2016; Olenik-Shemesh et al., 2017). In the online realm, internet self-efficacy represents the extension of self-efficacy into cyberspace. Internet self-efficacy refers to an individual’s confidence in their ability to utilize internet tools, engage in online activities, and attain predetermined objectives through these tools and actions (Eastin and LaRose, 2000). Compared with those who have lower internet self-efficacy, individuals with higher internet self-efficacy have more affirmative judgments of their internet capabilities, a heightened sense of control over the online environment, and a firmer belief in achieving desired outcomes through online actions. Consequently, when encountering others that face difficulties online, they are more confident in resolving issues and more likely to exhibit online altruistic behavior (Tao, 2013a; Pellas, 2014; Zhang, 2017).

Drawing from the bystander intervention model and pertinent empirical research, this study hypothesizes that in cyberbullying contexts, after bystanders form internet moral judgments and perceive a responsibility to intervene, individuals with higher internet self-efficacy will be more inclined to trust their abilities to identify specific intervention approaches than those with lower internet self-efficacy. Hence, they are more likely to participate in helping behavior. In summary, the third hypothesis in this study is brought forward:

*H3*: Internet self-efficacy moderates the relationship between internet moral judgment and bystander helping behavior in cyberbullying situations.

In the present study, building upon the empathy–altruism hypothesis, we investigate the connection between empathy and bystander helping behavior in the context of cyberbullying, thereby expanding this theory to encompass the cyberbullying domain. To date, few studies have examined the mediating mechanisms linking empathy and bystander helping behavior in cyberbullying from a cyber morality standpoint, such as internet moral judgment. Grounded in the dual-process model of morality, the current study explores the applicability of this model within cyberbullying settings, positing that empathy may not only directly stimulate bystander helping behavior but may also do so indirectly *via* the mediation of internet moral judgment. Moreover, there is a dearth of research concerning the moderating mechanisms between internet moral judgment and bystander helping behavior in cyberbullying. According to the bystander intervention model, the degree of internet self-efficacy may determine whether an individual ultimately engages in helping behavior, potentially offering vital insights for future effective intervention strategies. As such, this study investigates the moderating role of internet self-efficacy within the relationship between internet moral judgment and bystander helping behavior during cyberbullying incidents.

**Figure 1:**
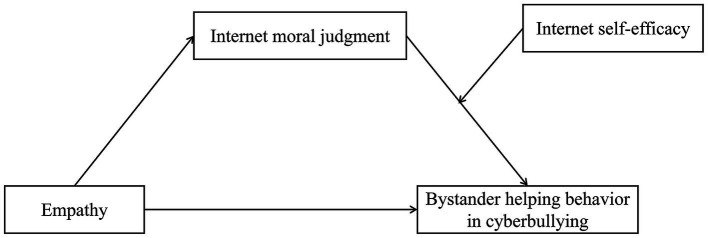
The model of the current study.

In summary, drawing from both theoretical and empirical perspectives, we formulated a moderated mediation model (Figure 1) described as follows: (1) Empathy may significantly and positively associate with bystander helping behavior in cyberbullying incidents; (2) Empathy may associate with bystander helping behavior in cyberbullying through the mediating effect of internet moral judgment; and (3) Internet self-efficacy may serve as a moderator between internet moral judgment and bystander helping behavior in cyberbullying situations.

## 2. Materials and methods

### 2.1. Participants

This study was approved by the Research Ethics Committee of the corresponding author’s institution. To ensure a diverse representation of youth groups and promote the generalizability of the findings, participants were recruited from three junior high schools in three provinces in China, namely, Hunan, Jiangxi, Guangdong. Before collecting the data, we obtained informed consent from all participants and their teachers. In addition, the principles of voluntary participation and confidentiality of the answers collected were emphasized to the participants. All participants completed the paper questionnaire in a classroom setting to guarantee the integrity of the assessment. Participants who did not report on the main study variables, and who clearly did not answer seriously (e.g., regular response) were excluded. This method of screening subjects has been widely used in many studies (Liu et al., 2022). In addition, small amounts of participant data were missing for individual items. The multiple imputation procedure in SPSS was used to impute missing data for individual missing items before conducting a scale computation. There were 360 (44.33%) seventh-graders and 452 (55.67%) eighth-graders. Ninth-graders did not participate in this survey because of the pressure of high school entrance exams. A total of 919 students participated in the survey, and 812 valid questionnaires were finally obtained. Approximately 50.62% of the sample was male, and the mean age was 12.70 years (SDage = 0.69).

### 2.2. Measures

#### 2.2.1. Empathy

We measured the participants’ empathy using the Basic Empathy Scale developed by Jolliffe and Farrington (2006). This self-report scale consists of 20 items. It includes two dimensions: cognitive empathy (e.g., “When someone is feeling ‘down’ I can usually understand how they feel”) and affective empathy (e.g., “After being with a friend who is sad about something, I usually feel sad”). Students rated the items on a five-point scale, ranging from 1 (strongly disagree) to 5 (strongly agree), and higher scores represented higher levels of empathy. The Basic Empathy Scale was originally validated on adolescents and was shown to have good reliability and validity (Jolliffe and Farrington, 2006). In the present study, the scale’s Cronbach’s α was 0.81.

#### 2.2.2. Internet moral judgment

We measured the participants’ internet moral judgment using the Internet Moral Judgment Questionnaire developed by Bi (2015). This self-report scale consists of 37 items. It includes four dimensions: understanding moral issues (e.g., “I do not think it’s right to repost untrue information on the Internet”), basis for moral judgment (e.g., “Searching for flesh of people involved in popular internet events should be banned”), reflection on moral reasoning (e.g., “When someone’s opinion differs from my own online, I first try to understand the basis of the other person’s opinion”), and behavioral plan (e.g., “I would not express my opinion about a hot topic on the Internet if I did not know what was going on”). Students rated the items on a five-point scale, ranging from 1 (completely not compliant) to 5 (completely compliant), with higher scores indicating higher online moral judgment. In this study, the scale’s Cronbach’s α was 0.91.

#### 2.2.3. Internet self-efficacy

We measured the participants’ internet self-efficacy using the Internet Self-Efficacy Questionnaire developed by Kou (2019). This self-report scale consists of 19 items. It includes three dimensions: information function (e.g., “I believe I can get online by clicking on the links”), email (e.g., “I believe I can send emails”), and other online actions (e.g., “I believe I can exchange information with other users in the forum”). Students rated the items on a five-point scale, ranging from 1 (completely not compliant) to 5 (completely compliant), with higher scores indicating higher levels of internet self-efficacy. In this study, the scale’s Cronbach’s *α* was 0.97.

#### 2.2.4. Styles of bystander intervention scale

We measured the participants’ bystander helping behavior in cyberbullying using the Styles of Bystander Intervention Scale developed by Moxey and Bussey (2019). This self-report scale consists of 15 items. It includes two dimensions: aggressive (e.g., “by making threats to the bully”) and constructive (e.g., “by encouraging the kid to report being picked on”) interventions. Answers were given on a five-point scale (1 = never, 5 = always), with higher scores indicating a greater tendency to engage in aggressive or constructive bystander interventions. In this study, the scale’s Cronbach’s α was 0.95.

#### 2.2.5. Covariates

This study controlled for gender, grade, cyberbullying perpetration, and cyberbullying victimization in the statistical analyses because previous research has shown that these variables may have an impact on bystander helping behavior in cyberbullying (Barlińska et al., 2013; Van Cleemput et al., 2014; Cao and Lin, 2015; Moxey and Bussey, 2019).

We measured adolescent cyberbullying perpetration by the 14-item perpetration subscale of the Revised Cyber Bullying Inventory (Chu and Fan, 2017). The subscale contains 14 items (e.g., “Posting false photos or information online to slander someone”). Each item was to assess how frequently adolescents performed incidences of cyberbullying perpetration in the past 6 months. Participants were asked to respond to the questions using a four-point scale (1 = never, 4 = more than three times), with higher scores indicating higher cyberbullying perpetration frequency. The scale has been shown to measure cyberbullying perpetration effectively in a sample of Chinese adolescents (Chu and Fan, 2017; Shi et al., 2021). In this study, this subscale’s Cronbach’s α was 0.71.

We measured adolescent cyberbullying victimization by the 14-item victimization subscale of the 0. Revised Cyber Bullying Inventory (Chu and Fan, 2017). The subscale contains 14 items (e.g., “I received false information online to defame me.”). Each item was to assess how frequently adolescents experienced incidences of cyberbullying victimization in the past 6 months. Participants were asked to respond to the questions using a four-point scale (1 = never, 4 = more than three times), with higher scores indicating higher cyberbullying victimization frequency. The scale has been shown to measure cyberbullying victimization effectively in a sample of Chinese adolescents (Chu and Fan, 2017; Liu et al., 2021). In this study, this subscale’s Cronbach’s α was 0.87.

### 2.3. Data analysis

First, IBM SPSS version 22.0 was used to calculate the descriptive statistics for the variables of interest, followed by bivariate correlations among these variables. Second, PROCESS macro (Hayes, 2013) was used to examine the mediating model *via* internet moral judgment (Model 4) and the moderating effect of internet self-efficacy (Model 14). Model 4 was applied for testing the mediating effect of internet moral judgment in the association between empathy and bystander helping behavior in cyberbullying. A bias-corrected bootstrapping with 5,000 samples was computed. If the 95% confidence interval (CI) did not include zero, it meant that the mediation effect was significant. If the mediating model was significant, we continued with the PROCESS to conduct the moderating analyses with Model 14. Participants’ gender, grade, cyberbullying perpetration, and cyberbullying victimization were controlled in the statistical analysis. All study variables were standardized in Model 4 and Model 14 before data analyses.

## 3. Results

### 3.1. Common method bias analysis

The data of the present study were all from self-report questionnaires. Therefore, we used Harman’s one factor test to examine common method bias. The results of the unrotated factor analysis showed that the first principal factor explained 18.43% of the variance, indicating that common method bias was not a serious problem in this study (Podsakoff et al., 2003).

### 3.2. Preliminary analyses

The descriptive statistics and correlation matrix for all study variables are provided in Table 1. Bivariate correlations showed that empathy was significantly positively associated with internet moral judgment, internet self-efficacy, and bystander helping behavior in cyberbullying (*p* < 0.001). Internet moral judgment was significantly positively associated with internet self-efficacy and bystander helping behavior in cyberbullying (*p* < 0.001). Internet self-efficacy was significantly positively associated with bystander helping behavior in cyberbullying (*p* < 0.001).

**Table 1:** Descriptive statistics and correlations between variables.

Variables	*M*	SD	1	2	3	4	5	6	7	8
Gender	–	–	–							
Grade	7.56	0.50	−0.03	1						
CV	16.13	4.51	−0.07	−0.01	1					
CP	14.73	2.09	−0.06	−0.04	0.46^***^	1				
Empathy	71.53	11.01	0.17^***^	0.05	−0.02	−0.05	1			
IMJ	98.09	22.98	0.14^***^	0.23^***^	0.10^**^	0.15^***^	0.15^***^	1		
ISE	64.55	22.81	−0.01	0.24^***^	0.06	0.07^*^	0.13^***^	0.32^***^	1	
BHBIC	27.55	13.61	−0.02	0.15^***^	0.20^***^	0.09^**^	0.18^***^	0.24^***^	0.30^***^	1

*N* = 860. M, mean; SD, standard deviation; CV, cyberbullying victimization; CP, cyberbullying perpetration; IMJ, internet moral judgment; ISE, internet self-efficacy; BHBIC, bystander helping behavior in cyberbullying. Gender was dummy coded such that male = 1 and female = 0. ^*^p < 0.05; ^**^p < 0.01; ^***^p < 0.001.

### 3.3. Testing for mediation effect

Results showed the total effect of empathy and bystander helping behavior in cyberbullying at 0.17, *p* < 0.001, 95% CI = [0.10, 0.23], supporting H1.

Empathy was significantly and positively related to adolescents’ internet moral judgment (*b* = 0.13, *p* < 0.001), which in turn was significantly and positively related to bystander helping behavior in cyberbullying (*b* = 0.21, *p* < 0.001). Moreover, the residual direct effect of empathy on bystander helping behavior in cyberbullying was still significant (*b* = 0.14, *p* < 0.001). The indirect effect of empathy on bystander helping behavior in cyberbullying *via* internet moral judgment was 0.03, SE = 0.01, 95% CI = [0.01, 0.05]. Empirical 95% CI did not consist of zero, indicating that adolescents’ internet moral judgment mediated the association between empathy and bystander helping behavior in cyberbullying, thus verifying H2.

### 3.4. Moderated mediation effect analysis

**Figure 2:**
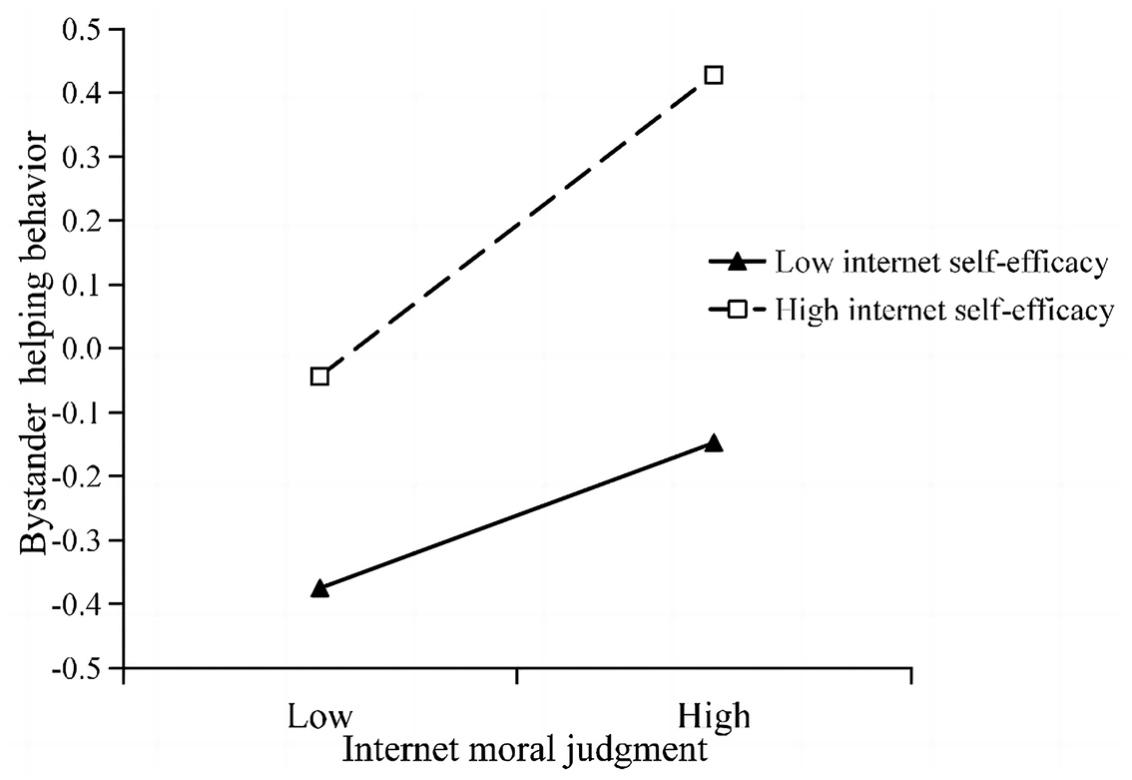
Plot of the relationship between internet moral judgment and bystander helping behavior in cyberbullying at two levels of internet self-efficacy.

As shown in Table 2, the interaction terms of internet moral judgment and internet self-efficacy was significantly and positively related to bystander helping behavior in cyberbullying (*β* = 0.06, *p* < 0.05). For descriptive purposes, we plotted explored internet moral judgment on bystander helping behavior in cyberbullying, separately for low and high levels of internet self-efficacy (1 SD below the mean and 1 SD above the mean, respectively; see Figure 2). Simple slope tests showed that internet moral judgment was significantly associated with bystander helping behavior in cyberbullying for adolescents with high-level internet self-efficacy and low-level internet self-efficacy, but the association between internet moral judgment and bystander helping behavior in cyberbullying was stronger for adolescents with high levels of internet self-efficacy (bsimple = 0.23, *p* < 0.001) than for adolescents with low levels of internet self-efficacy (bsimple = 0.11, *p* < 0.01). Furthermore, the conditional indirect effects were tested (at the following levels of the moderator: M – 1 SD, M, and M + 1 SD). As shown in Table 3, among individuals with low (M – 1 SD), medium (M) and high (M + 1 SD) levels of internet self-efficacy, internet moral judgment was in all cases significantly and positively related to bystander helping behavior in cyberbullying, thus supporting H3.

**Table 2:** Testing the moderating effect of internet self-efficacy on the relation between internet moral judgment and bystander helping behavior in cyberbullying.

Predictors	Outcome (IMJ)				Outcome (BHBIC)			
	*β*	SE	*t*	95%CI	*β*	SE	*t*	95%CI
Gender	0.27^***^	0.07	4.03	[0.14, 0.41]	−0.08	0.07	−1.15	[−0.20, 0.05]
Grade	0.46^***^	0.07	6.85	[0.33, 0.59]	0.13^*^	0.07	1.98	[0.001, 0.26]
CV	0.05	0.04	1.31	[−0.02, 0.12]	0.19^***^	0.04	5.30	[0.12, 0.26]
CP	0.15^***^	0.04	4.11	[0.08, 0.23]	−0.02	0.04	−0.52	[−0.09, 0.05]
Empathy	0.13^***^	0.03	3.92	[0.07, 0.20]	0.13^***^	0.03	3.90	[0.06, 0.19]
IMJ					0.17^***^	0.04	4.82	[0.10, 0.24]
ISE					0.23^***^	0.03	6.52	[0.16, 0.29]
IMJ × ISE					0.06^*^	0.03	2.32	[0.01, 0.11]
*R*^2^	0.12				0.19			
*F*	21.87^***^				22.80^***^			

*N* = 860. ^*^p < 0.05. ^***^p < 0.001.

**Table 3:** The conditional indirect effects of internet self-efficacy.

Internet self-efficacy	Effect	Boot *SE*	Boot LLCI	Boot ULCI
M – 1 SD	0.015	0.006	0.005	0.028
M	0.023	0.007	0.010	0.039
M _+_ 1 SD	0.031	0.010	0.013	0.053

SE, standard error; CI, confidence interval; LL, lower limit; UL, upper limit.

## 4. Discussion

This study built on previous empirical studies and theories to clarify not only the relationship between empathy and bystander helping behavior in cyberbullying, but also reveal the mediating role of internet moral judgment and the moderating role of internet self-efficacy, thus supporting the hypotheses. The findings of this study have both theoretical and practical significance, as they enhance the understanding of the connection between empathy and bystander intervention in cyberbullying situations and provide empirical support for designing intervention measures from a bystander’s perspective.

### 4.1. The relationship between empathy and bystander helping behavior in cyberbullying situations

The total effect model demonstrates that empathy significantly and positively associates with bystander helping behavior in cyberbullying. When witnessing cyberbullying, individuals with higher levels of empathy are more inclined to assist the victims, thus validating H1. Consistent with previous research findings, empathy was found to be associated with bystander helping behavior in cyberbullying situations (Barlińska et al., 2013; Machackova et al., 2015).

Empathy is a pro-social personality trait (Jing et al., 2017b); when bystanders notice cyberbullying events, those with higher empathy levels can better perceive the emotional responses of the victims and understand the harm inflicted by the cyberbullying acts, which in turn motivates bystanders to engage in helping behaviors (Schultze-Krumbholz et al., 2019; Ji, 2021). Particularly in an online environment, bystanders with high empathy levels can empathize with the pain of victims in the absence of empathic cues, thus increasing their positive bystander behaviors (Owusu and Zhou, 2015). Additionally, individuals with heightened empathy are more prone to perspective-taking, imagining themselves in the same predicament as the cyberbullying victims and recognizing the need for help from others (Wang, 2014). Consequently, under the guidance of these emotions, empathetic bystanders are more willing to help victims (Freis and Gurung, 2013; Jing et al., 2017a).

### 4.2. The mediating role of internet moral judgment

This study’s results indicate that empathy is associated with bystander helping behavior in cyberbullying scenarios through the partial mediation of internet moral judgment, thereby validating H2. The results of this study support the dual-process model of morality. Moral emotions, such as empathy, is not only directly associated with moral behavior like bystander helping behavior in cyberbullying, but also indirectly associated with moral behavior through moral reasoning and moral judgment (Chaiken and Trope, 1999; Haidt, 2001; Zhao, 2016; Wu et al., 2017). This finding helps explain the mediating mechanism between empathy and bystander helping behavior in cyberbullying.

On the one hand, this study’s results are consistent with previous findings that a positive association exists between empathy and moral judgment (Decety and Cowell, 2014; Patil and Silani, 2014). The moral judgment of highly empathetic individuals is primarily affected by the perceived degree of harm inflicted upon victims. In situations involving moral transgressions, those with high empathy are often most sensitive to others’ pain and suffering, which, in turn, influences their perspectives and assessments of such morally transgressive incidents. A more profound perception of harm results in a higher moral judgment score (Wang and Yang, 2021). However, in the online context, the limited cues available make it challenging for bystanders to observe the victims’ reactions and understand the actual harm resulting from cyberbullying (Kraft, 2011; Song and Oh, 2018). Still, individuals with strong empathetic abilities can resonate with the victims’ pain even in environments lacking these cues (Ji, 2021). This heightened sensitivity enables them to recognize the urgency of the situation and helps them make more accurate moral judgments in an online environment where non-compulsory moral standards are diminished (Fan, 2012).

On the other hand, the results are consistent with previous studies that have shown that individuals with higher moral judgment abilities are more likely to exhibit altruistic prosocial behaviors (Guo, 1999; Li, 2005; Eisenberg and Spinrad, 2014; Patrick et al., 2018). In online contexts, Li (2015) also found a significant positive correlation between college students’ internet moral judgment abilities and online altruistic behavior. Bystanders with higher levels of internet moral judgment are more likely to perceive cyberbullying incidents as immoral, thus igniting their moral awareness and responsibility to engage in helping behaviors, ultimately leading to increased positive helping behaviors (Fan, 2012; Owusu and Zhou, 2015). As such, bystanders’ empathy affects their helping behaviors in cyberbullying incidents by improving their internet moral judgment.

### 4.3. The moderating role of internet self-efficacy

Further, this study discovered that the mediating effect of internet moral judgment was moderated by internet self-efficacy. Specifically, the positive association between internet moral judgment and bystander helping behavior in cyberbullying situations was progressively enhanced as internet self-efficacy levels increased, confirming H3.

These findings align with the promotion hypothesis within the “protective factor–protective factor model,” which suggests that one protective factor may amplify the predictive effect of another protective factor on the outcome variable (Bao et al., 2013). In this study, both internet moral judgment and internet self-efficacy served as protective factors for bystander helping behavior in cyberbullying cases. These two protective factors interacted, which had an impact on the developmental outcomes—with internet self-efficacy effectively boosting the association between internet moral judgment and bystander helping behavior in cyberbullying scenarios.

This may be because, in the bystander intervention model (Latané and Darley, 1970), individuals evaluate their capacity to intervene between ascertaining their responsibility to help through internet moral judgment and actually engaging in helping actions (Huang et al., 2019). According to Bandura’s motivational theory, individuals will only undertake certain behaviors if they have high self-efficacy, meaning they trust their ability to execute the behavior and manage its consequences (Bandura, 1977; Wang and Yang, 2021). In cyberbullying incidents, even if bystanders experience a sense of responsibility to help through internet moral judgment, those with low internet self-efficacy might question their ability to provide meaningful help (Tao, 2013b). They may lack confidence in their capacity to complete online tasks, worry about their insufficient online operational skills, or be uncertain about how to halt cyberbullying effectively or console victims using online methods (Hsu and Chiu, 2004). This hesitation may decrease the likelihood of helping cyberbullying victims (Tao, 2013b; Zhang, 2017). Conversely, bystanders with higher levels of internet self-efficacy possess a more optimistic evaluation of their capabilities (Li, 2004). Confident about using the Internet, they gain a stronger sense of control in the online environment (Liu, 2015). Hence, when carrying out intervention actions, they are more capable of supporting victims and halt bullying behaviors in cyberbullying incidents (Zhang, 2017).

Thus, in cyberbullying situations, internet self-efficacy moderates the relationship between internet moral judgment and bystander helping behavior, signifying that bystanders with high levels of internet moral judgment are more inclined to aid cyberbullying victims when they have high internet self-efficacy; by contrast, willingness to help is comparatively lower when internet self-efficacy is low.

### 4.4. Limitations and implications

This study has several limitations that should be addressed in future research. First, this study is a cross-sectional study rather than a rigorously designed experiment. Hence, it cannot infer a causal relationship. Second, the data collected were based on self-reports from adolescents, which may be subject to social desirability bias and lack ecological validity. Future studies could incorporate situational experiments to gather more objective and comprehensive data. Third, this study solely examined the impact of individual factors on bystander helping behavior, without considering environmental factors or the interaction between individual and environmental factors. Finally, the empathy and moral judgment in cyberspace measures used in this study encompass various dimensions, and future research could further investigate the performance of these factors across these dimensions to determine their distinct roles.

Despite these limitations, the study results have important theoretical and practical implications. From a theoretical perspective, the findings provide further validation for the empathy–altruism hypothesis in the context of cyberbullying by revealing a significant positive correlation between empathy and bystander helping behavior in cyberbullying among adolescents. Additionally, few studies have investigated online helping behavior from a moral psychology perspective (Wu et al., 2017). This study focused on the moral behavior of bystanders in cyberbullying situations, explored the relationship between moral emotion, such as empathy, and the bystander helping behavior in cyberbullying, and further revealed the mediating role of internet moral judgment between empathy and bystander helping behavior in cyberbullying. The results verified the applicability of the moral dual-processing model in bystander helping behavior in cyberbullying. Furthermore, the bystander intervention model, as a processual model, only presents stage changes in bystander helping behavior (Huang et al., 2019). The moderated mediation model examined in this study revealed the mechanisms of empathy, internet moral judgment, and internet self-efficacy on bystander helping behavior in cyberbullying, which not only validated the bystander intervention model in the context of cyberbullying among adolescents, but also showed the underlying mechanisms connecting the stages of the bystander intervention model, thus contributing to a deeper understanding of the individual psychological processes that shape bystander helping behavior in cyberbullying situations.

From a practical standpoint, the results offer guidance on promoting bystander intervention through educational measures in response to cyberbullying incidents and present new perspectives for educators to reduce the prevalence and negative impact of cyberbullying from the bystander’s viewpoint. First, the study identified a positive correlation between empathy and bystander helping behavior in cyberbullying. In adolescent education, teachers can work to improve students’ empathy levels by organizing activities such as reflective discussions or role-playing exercises to enable students to comprehend the detrimental effects of cyberbullying on victims (Machackova and Pfetsch, 2016) and increase their likelihood of offering help. Second, the study found that empathy is associated with bystander helping behavior in cyberbullying through internet moral judgment. This insight emphasizes the importance of reinforcing moral education for young people in the digital realm and fostering their moral cognition and judgment to discern right from wrong, recognize the harm caused by cyberbullying, and become more inclined to help victims. Lastly, the study revealed that internet self-efficacy moderates the mediating role of internet moral judgment between empathy and bystander helping behavior. Therefore, in an era where people increasingly communicate and interact online (Kamalpour et al., 2020), teachers should not only guide students in using the Internet appropriately but also enhance their online skills and confidence. By doing so, bystanders in cyberbullying situations will feel more empowered to engage in helping behaviors, ultimately reducing the frequency and negative consequences of cyberbullying.

## 5. Conclusion

This study investigated the relationship between empathy and cyberbullying bystander helping behavior, and explored the mediating role of internet moral judgment and the moderating role of internet self-efficacy. Our key findings are as follows: (1) Empathy is significantly and positively associated with bystander helping behavior in cyberbullying; (2) Internet moral judgment partially mediates the relationship between empathy and bystander helping behavior; and (3) The second half of the mediating effect of internet moral judgment is moderated by internet self-efficacy. In particular, the association between internet moral judgment and helping behavior is greater for bystanders with higher internet self-efficacy compared with those that have lower internet self-efficacy.

## Data availability statement

The raw data supporting the conclusions of this article will be made available by the authors, without undue reservation.

## Ethics statement

The studies involving human participants were reviewed and approved by the Research Ethics Committee of the Central China Normal University. Written informed consent to participate in this study was provided by the participants’ legal guardian/next of kin.

## Author contributions

YH: conceptualization, methodology, and writing – review and editing. TZ: conceptualization, investigation, visualization, and writing – review and editing. H-fS: conceptualization, visualization, and writing – review and editing. C-yF: conceptualization, methodology, funding acquisition, project administration, and supervision. All authors contributed to the study and approved the submitted version of the article.

## Funding

This work was supported by the Research Program Funds of the Collaborative Innovation Center of Assessment for Basic Education Quality at Beijing Normal University (Grant number 2022–04-009-BZPK01).

## Conflict of interest

The authors declare that the research was conducted in the absence of any commercial or financial relationships that could be construed as a potential conflict of interest.

## Publisher’s note

All claims expressed in this article are solely those of the authors and do not necessarily represent those of their affiliated organizations, or those of the publisher, the editors and the reviewers. Any product that may be evaluated in this article, or claim that may be made by its manufacturer, is not guaranteed or endorsed by the publisher.
